# Usage of Modified Makuuchi Incision for Surgical Management of Complex Renal and Adrenal Lesions

**DOI:** 10.7759/cureus.11012

**Published:** 2020-10-18

**Authors:** Sri Harsha Bokka, Sreerag Sreenivasan Kodakkattil, Ramanitharan Manikandan, Dorairajan Lalgudi Narayanan, Hemachandren M, Sidhartha Kalra, Pottakkat Biju

**Affiliations:** 1 Urology and Renal Transplantation, Jawaharlal Institute of Postgraduate Medical Education and Research (JIPMER), Puducherry, IND; 2 Cardiothoracic and Vascular Surgery, Jawaharlal Institute of Postgraduate Medical Education and Research (JIPMER), Puducherry, IND; 3 Surgical Gastroenterology, Jawaharlal Institute of Postgraduate Medical Education and Research (JIPMER), Puducherry, IND

**Keywords:** modified makuuchi incision, radical nephrectomy, adrenalectomy, liver resection, ivc thrombectomy

## Abstract

Background: Modified Makuuchi (MM) incision is less popular among the urological fraternity as Chevron, subcostal, flank, and midline incisions are commonly used for most of the complex renal and adrenal conditions. We present our experience and report the outcomes of patients operated using this incision.

Materials and methods: The records of patients who underwent open surgery for upper abdominal urological conditions using MM incision over the last five years in our department were retrospectively reviewed. Patient demographics, laterality of the lesion, size of the lesion, level of inferior vena caval (IVC) thrombus, intraoperative blood loss, local tumor invasion, need for concomitant hepatectomy, need of diaphragmatic resection, use of self-retaining retractors, operative time, hospital stay, wound-related complications, and readmissions were analyzed.

Results: Some 18 patients underwent open surgery by this incision for various complex renal and adrenal conditions during the study period. Patients included those with large upper pole renal and adrenal masses, renovascular conditions like renal artery aneurysm, renal/adrenal masses with liver and diaphragmatic infiltration requiring hepatectomy, diaphragmatic resections, or IVC thrombectomy. The mean size of renal and adrenal masses was 13.8 (±6.3) cm, mean operative time was 370 (±210.6) minutes, mean blood loss was 1124 (±990.3) mL, and mean hospital stay was 11.65 (±13.2) days. Four patients had surgical site infection (SSI) and one had readmission.

Conclusion: The MM incision can be widely adapted for complex renal and adrenal surgeries and should become a part of the various commonly used incisions by urologists.

## Introduction

Complex renal surgeries are usually operated by flank, Chevron, subcostal, midline, and thoracoabdominal incisions whenever an open surgery is deemed mandatory [1]. Similar incisions are also described in the context of complex adrenal surgeries and also when there is a suspicion of malignancy [2-4]. Another incision which can be used in dealing with the aforementioned conditions is modified Makuuchi (MM) incision which is yet to gain popularity in urology. The Makuuchi incision consists of an upper vertical midline incision that originates from the xiphoid process, extends caudally up to 5 cm above the umbilicus, and then curves laterally as a J along the ninth intercostal space to end at the posterior axillary line [5]. This was later modified by Chang, in which the vertical limb remained the same whereas the horizontal limb curves laterally as a reverse L in parallel to the anatomic abdominal skin fold at the level of umbilicus and also along the dermatomal distribution of the nerves to end at the midpoint between the lowest rib and the anterosuperior iliac spine [6]. The utility of these incisions has been described in great depth in a wide variety of foregut and hepatobiliary surgeries [6-7] but to a limited extent in upper abdominal urological surgeries. We routinely use MM incision for its unique advantages like the unparalleled exposure of great vessels in their entirety along with the lesion of interest and liver mobilization which greatly helped us in dealing with a variety of renal, adrenal, and renovascular conditions. The aim of the present study is to report the utility of MM incision in the management of complex upper abdominal urological surgeries and its advantages over the conventional incisions.

## Materials and methods

**Figure 1:**
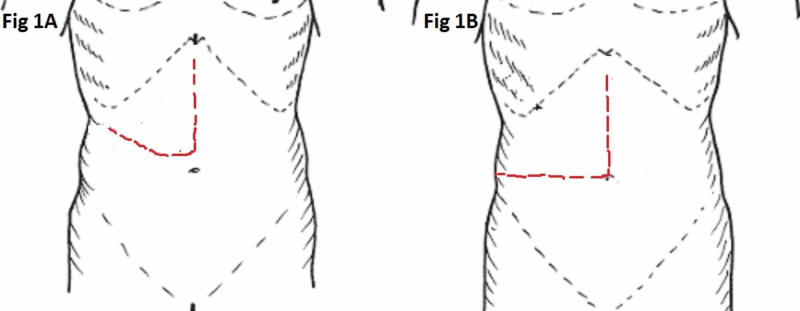
Makuuchi and MM incisions. A: Conventional Makuuchi incision. B: Modified Makuuchi incision MM, Modified Makuuchi

The medical records of all 18 patients who underwent complex open renal and adrenal surgeries via MM incision (Figure 1) from January 2015 to January 2020 in the Department of Urology and Renal Transplantation in Jawaharlal Institute of Postgraduate Medical Education and Research (JIPMER), Puducherry, India were retrospectively reviewed. Preoperative parameters including patient’s age, gender, laterality of pathologic condition, intraoperative parameters like MM incision with or without the need of extension as per the requirement, operative time, types of retractors employed, resection of adjacent organs, and blood loss were determined. The total number of cases with surgical site infection (SSI), the ones requiring antibiotic therapy and secondary suturing, hospital stay, and readmission rates for wound-related complaints were noted. Novick’s classification system was used to define the level of inferior vena caval (IVC) thrombus where radical nephrectomy (RN) with IVC thrombectomy was performed [1].

All continuous variables like age, size of the lesion, operative time, blood loss, and hospital stay were expressed as mean with standard deviation, and categorical variables like laterality of lesion, diagnosis, surgery performed, wound-related complications, and readmissions were expressed as the frequency with percentages. SPSS® 23.0 software (IBM Corp, Armonk, NY, USA) was used for statistical analysis. Wound-related complications were expressed using Clavien Dindo Classification.

## Results

**Figure 2:**
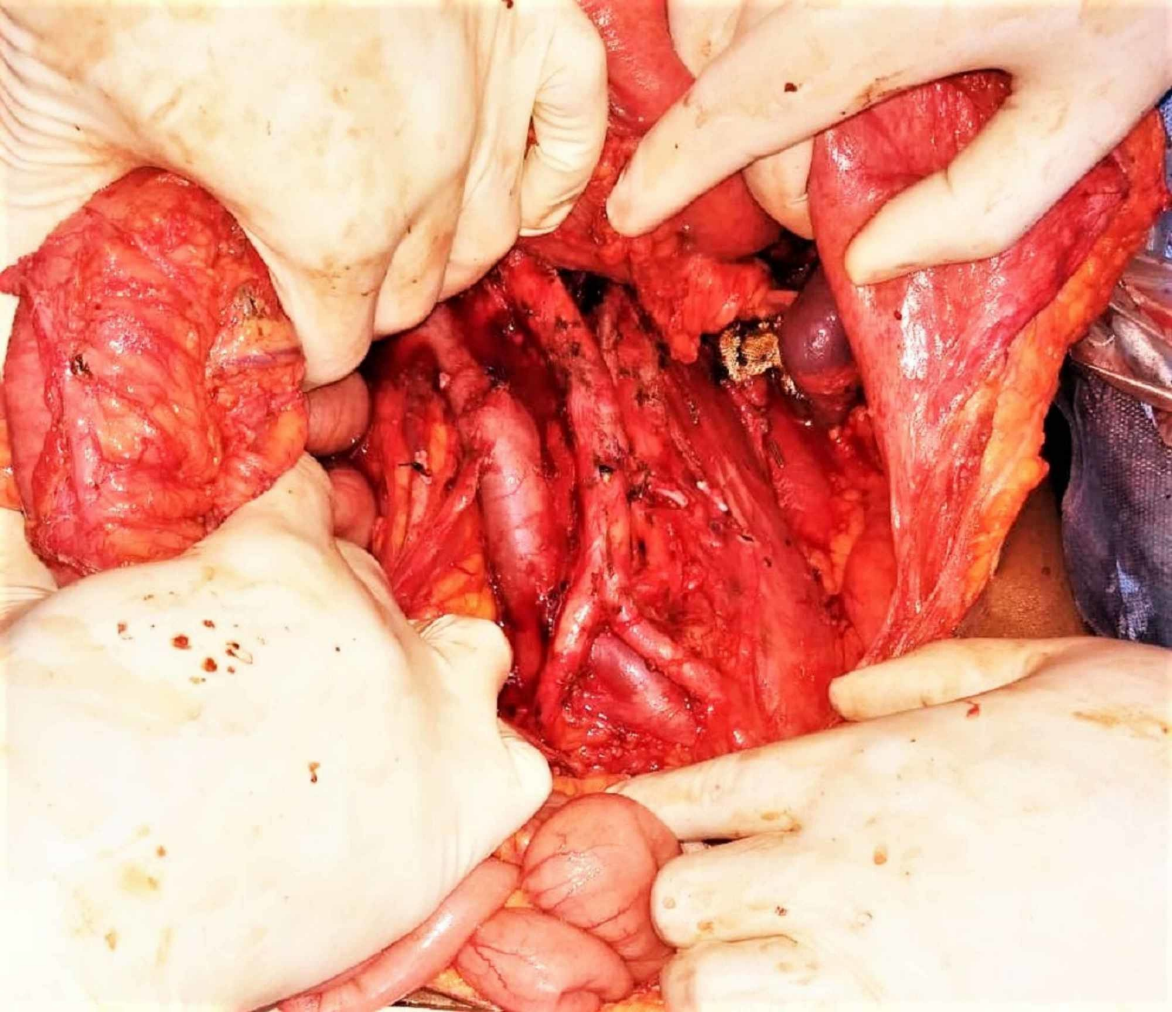
Left MM incision with exposed inferior vena cava and aorta along the entirety-post left radical nephrectomy status. MM, Modified Makuuchi

**Figure 3:**
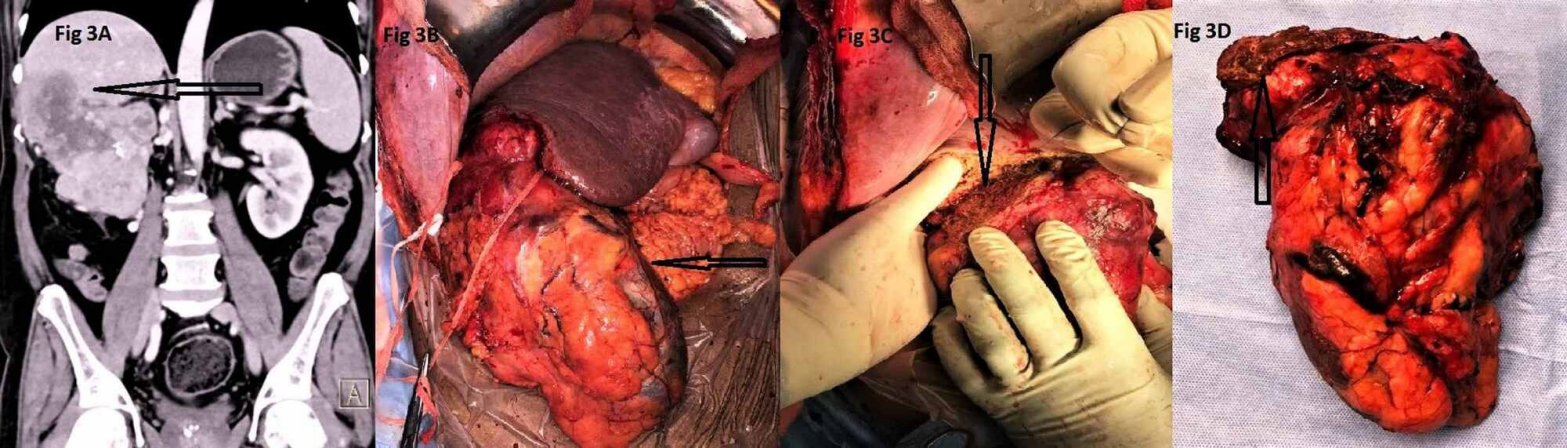
A: Contrast enhanced CT showing right renal mass with arrow indicating liver infiltration. B: Intraoperative picture with arrow showing completely mobilized right kidney. C: Intraoperative picture with arrow showing liver resection in process. D: RN specimen with arrow indicating the resected portion of liver at the upper pole of the kidney. RN, radical nephrectomy

Operations performed in these 18 patients included three RNs (mean size - 18.3 cm in maximum dimension) (Figure 2), two RNs with nonsegmental liver resection (Figure 3), and seven RNs with IVC thrombectomy. One emergency upper pole partial nephrectomy was done for renal abscess with infarction of upper pole of left kidney along with resection and anastomosis of jejunal and descending colon perforation. The final histopathological diagnosis in this case was suggestive of mucormycosis. Similarly, one case of right adrenalectomy with en bloc excision of adjacent structures for infiltration and another case of excision of tumor bed recurrence in a case of renal cell carcinoma (RCC) with RN performed in the past were dealt with the help of MM incision. In a case of 4 cm left renal artery aneurysm at the bifurcation of main renal artery into segmental arteries along with massive splenomegaly and portal hypertension, nephrectomy for bench reconstruction of the aneurysm with splenectomy and splenorenal shunt via MM incision and auto-transplantation of concerned kidney subsequently via Gibson’s incision was performed. Two cases of adrenalectomy were performed where one was a case of 12 cm x 12 cm adrenal mass and the other one was a case of adrenal pheochromocytoma of size 6 cm x 6 cm in a patient with multiple endocrine neoplasia (MEN) where along with excision of adrenal mass, a biopsy was also taken from a lesion in the head of the pancreas through the same incision.

 

**Figure 4:**
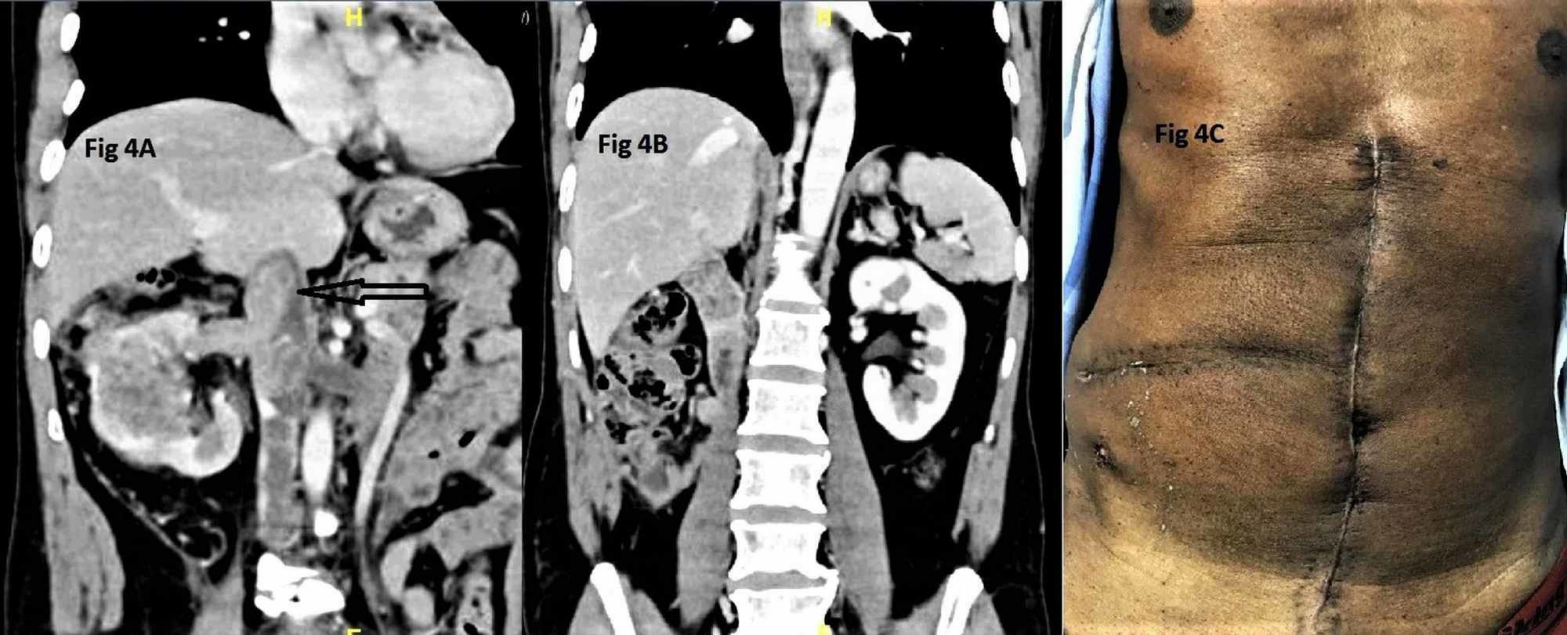
A: Preoperative contrast enhanced CT showing mass in right kidney with arrow indicating enhancing tumor thrombus in inferior vena cava and long bland thrombus in infra renal vena cava. B: Three months postoperative CT showing no evidence of recurrence. C: Healthy double Makuuchi scar after three months of surgery.

A special mention of RCC with IVC thrombus is needed here. One case of level I thrombus, three cases of level II, two cases of level III, and one case of level IV thrombus were operated. Surgeries of RCC with level I and level II thrombi were successfully performed by MM incision without any additional maneuvers. A case of level III IVC thrombus had a long bland thrombus in the infrarenal portion of IVC extending up to common iliac veins and this patient underwent IVC thrombectomy of tumour thrombus after complete liver mobilization by Piggyback technique, Pringle’s maneuver, control of extensive peritumoral collaterals and IVC interruption via double Makuuchi incision. Double Makuuchi incision consisted of a vertical limb extended from the angle of MM incision below the umbilicus up to the pubic symphysis (Figure 4).

Another patient had level IIId IVC thrombus with intrapericardial extension. Complete mobilization of the liver with opening of the central diaphragm tendon and the pericardium exposed the supradiaphragmatic and intrapericardial IVC thereby permitting IVC thrombus removal without a median sternotomy. The case of RCC with level IV IVC thrombus underwent nephrectomy and liver mobilization via MM incision and thrombectomy from the right atrium via median sternotomy by cranial extension of the vertical limb of MM incision.

**Figure 5:**
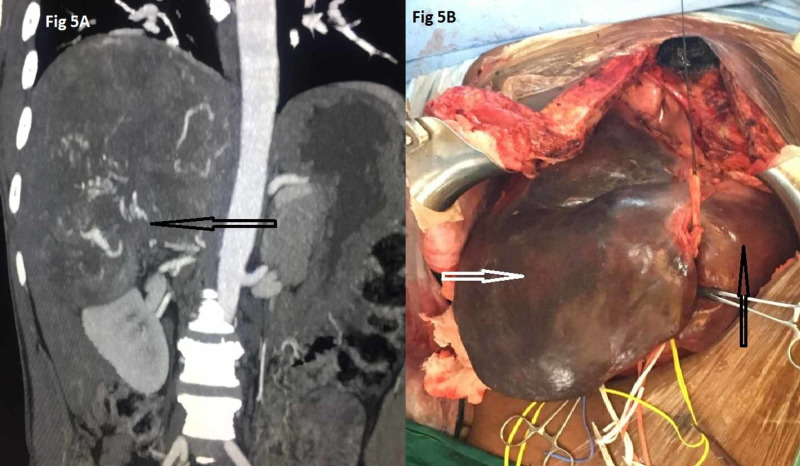
A: CT image showing right adrenal mass with infiltration of right kidney and indistinct planes with liver and extensive tumor vascularity. B: Intraoperative picture of the same case where median sternotomy was done for supra diaphragmatic IVC control and veno-venous bypass. Black arrow indicates left lobe of liver and white arrow indicates adrenal mass with extensive infiltration of right lobe of liver. IVC, inferior vena caval

Similarly, a case of 30 cm x 30 cm right adrenal lesion with right renal, hepatic, diaphragmatic, and IVC infiltration was successfully operated via MM incision where adrenalectomy, en bloc right nephrectomy, resection of right hemidiaphragm, right hepatectomy, resection of an infiltrated segment of IVC followed by its graft reconstruction were done. Here, as supra-diaphragmatic IVC control was needed in view of hepatic venous involvement, a median sternotomy was done by cranial extension of the vertical limb of MM incision, and the procedure was completed successfully after veno-venous bypass (Figure 5).

We routinely use self-retaining retractors like Thompson or Bookwalter to maximize the exposure whenever a MM incision is employed.

Patient demographics, diagnosis, surgery performed, various maneuvers employed, and outcomes are listed in Table 1. Overall, the mean size of renal and adrenal masses was 13.8 (±6.3) cm, mean operative time was 370 (±210.6) minutes, mean blood loss was 1124 (±990.3) mL, and mean hospital stay was 11.65 (±13.2) days. There were four cases of class 1, one case of class 3A, and no class 3B or above complications in our study. The only readmission in our experience was for a case of SSI which was managed with IV antibiotics and secondary suturing under local anesthesia. None of the patients in our study complained of any paraesthesias along the incision. None developed incisional hernia.

**Table 1:** Patient demographics, tumor characteristics, operative parameters, and outcomes. SD, standard deviation; IVC, inferior vena caval; SSI, surgical site infections; IV, intra venous; RN, radical nephrectomy

Variable	Subvariable	Value
Mean age (SD)		52 years (± 12.2)
Mean size (SD)		13.8 cm (±6.3)
Laterality of the lesion	Right	14 (77.8%)
	Left	4 (22.2%)
Sex	Male	14 (77.8%)
	Female	4 (22.2%)
Diagnosis	Renal mass	12 (66.7%)
	Adrenal pathology	3 (16.7%)
	Recurrence in tumor bed after RN	1 (5.5%)
	Renal abscess with infarction of upper pole with jejunal and descending colon perforation	1 (5.5%)
	Renal artery aneurysm with portal hypertension	1 (5.5%)
Renal mass with IVC thrombus (n=7)	Level I	1 (14.3%)
	Level II	3 (42.8%)
	Level III	2 (28.6%)
	Level IV	1 (14.3%)
Surgery performed	RN with IVC thrombectomy	7 (38.9%)
	RN	3 (16.7 %)
	RN with nonsegmental liver resection	2 (11.1%)
	Adrenalectomy	1 (5.5%)
	Adrenalectomy for pheochromocytoma with biopsy of pancreatic head mass	1 (5.5%)
	Adrenalectomy + en bloc right nephrectomy + resection of right hemidiaphragm with mesh repair + right hepatectomy + resection of infiltrated segment of IVC and graft reconstruction	1 (5.5%)
	Excision of tumor bed recurrence along with serosal nodules over stomach and diaphragm	1 (5.5%)
	Partial nephrectomy with resection and anastomosis of jejunum and descending colon	1 (5.5%)
	Nephrectomy for bench reconstruction of renal artery with splenectomy and spleno-renal shunt	1 (5.5%)
Manuevers employed in IVC thrombectomy	IVC interruption	1
	Supra-hepatic IVC clamping	2
	Transabdominal intrapericardial IVC control by opening of central tendon of diaphragm	1
	Complete liver mobilization by Piggyback technique	4
Mean operative time (SD)		369.7 min (±210.6)
Mean blood loss (SD)		1123.5 mL (±990.3)
Mean hospital stay (SD)		11.6 days (±13.2)
Clavein Dindo Complications	Class I	4 (23.6%)
	Class IIIA	1 (5.8%)
Readmissions	For SSI requiring IV antibiotics and secondary suturing	1 (5.8%)

## Discussion

The original description of Makuuchi incision by Masatoshi Makuuchi in 1993 seems to be similar to Giuliani’s incision which is described as an anterolateral transabdominal approach for renal tumors providing good visualization and good access to the renal pedicle, as well as good exposure caudally as far as the aortic bifurcation and cranially as far as the diaphragm [8]. A modification to the original description was made by Giberti and Schenone, and the authors concluded in their study that their modified anatomical incision compared to the conventional technique eliminated permanent functional deficits and hypotonia of the abdominal wall in their study subjects [9].

The traditionally used incisions like flank, subcostal, chevron and midline by the urologist for renal and adrenal pathologies are sufficient in most of the situations, but, for very large tumors or surgeries requiring the manipulation of great vessels and other structures like spleen, liver and pancreas, they have their own shortcomings [10]. The flank approach does not provide sufficient exposure for very large tumors. A transabdominal midline incision may provide reasonable exposure but suffers from a telescopic effect which can be a disadvantage, especially if the adjacent organs such as the spleen, liver, pancreas, and, occasionally, the stomach are not fully mobilized and may lead to injury of local structures. A thoracoabdominal incision provides excellent exposure but, has more morbidity because of the need for a chest tube and greater postoperative analgesia [10-11]. The MM incision has proved to be a gamechanger in our experience as it has successfully overcome all these drawbacks.

The utility of Makuuchi incision in major upper abdominal surgeries is described in great depth by Pandit et al. In their experience of 144 cases of upper gastrointestinal and hepato-pancreatico-biliary surgeries, the authors have described that the incision provided superior operative ergonomics, ease of entry and closure, ease of extension whenever required, maintained muscle integrity and resulted in less postoperative pain [7].

The available literature of Makuuchi incision in renal and adrenal tumors, though sparse, has come up recently. In a study of 29 cases of renal tumors described by Ali Atan and colleagues, the authors performed 26 RNs and three partial nephrectomies. The mean tumor size was 11.3 cm roughly similar to our study parameter (12.8 cm). The authors have also described 11 cases of RCC with venous tumor thrombus operated through this incision [12]. Though the maximum level of IVC thrombus described in their study is level II, we had no problem in tackling a level III tumor with MM incision. Not only we were able to do a complete liver mobilization, but also a supradiaphragmatic, intrapericardial control of IVC was taken by opening of the central tendon of the diaphragm via MM incision.

Similarly, Ruffolo et al., described their experience of 41 cases of open adrenalectomy via Makuuchi incision. Though the mean tumor size in their study is 8 cm, 14 patients required multi-visceral resection, including an en bloc resection of a locally invasive 18-cm pheochromocytoma along with a large hepatic wedge, nephrectomy, and an 8-cm segment of inferior vena cava, all done successfully via this incision. The mean operative time and the mean hospital stay reported by the authors were 322 minutes and 6 days respectively. We have operated only three cases of adrenalectomy via MM incision and hence, cannot compare our results with this study [13]. Nevertheless, the results of the study are quite encouraging and support our inclination towards MM incision.

The description of MM incision by Chang for a wide variety of 137 cases of hepatic, gastric, pancreatic and other retroperitoneal tumors sparked the idea of using it for complex renal and adrenal surgeries in us as it has been rightly described to facilitate safe exposure of inferior vena cava, aorta, adrenal gland, and right kidney [6].

In MM incision, the abdominal flap created can be totally lifted off supero-laterally and suture fixed. The exposure after this manuever is maximized with the use of a self-retaining retractor, thus, leading to a panoramic view of kidney and adrenals and aiding in complete liver mobilization whenever needed. Posterior segments of the right hemi-liver (segments 6 and 7) are the commonly involved liver segments in renal and liver tumors and access to these segments is difficult with common incisions in urology. Situations in which renal or adrenal tumor is infiltrating the liver segment, MM incision helps to mobilize along the retro-renal plane and perihepatic plane so that the renal-adrenal-liver complex is mobilized anteriorly, practically delivering the complex to the midline. This manuever along with a 15-30-degree left tilt to the operating table makes IVC clearly visible in its entire length along with renal, adrenal, and hepatic veins. This helped us in not only performing nonsegmental liver resections but also right hemi-hepatectomy in a case of large adrenal mass where along with the latter, diaphragmatic resection and subsequent reconstruction were accomplished successfully. Moreover, in case of the renal mass infiltrating colon or its mesentery, finding a plane between the two structures, especially at the lower pole of the kidney requires an adequate exposure which is feasible with MM incision. In our experience, whenever supra-hepatic IVC control was needed, it was achieved comfortably after liver mobilization adapting the techniques of orthotopic liver transplantation described by Ciancio et al., via MM incision [14]. All the aforementioned surgical techniques are difficult to perform using other traditional incisions because of the difficulties that are anticipated as described in the literature [10-11].

We have found MM incision convenient for large renal tumors (up to 20 cm in size), large adrenal tumors (up to 30 cm in size), RCC with IVC thrombus even up to level III, and RCC with liver infiltration. In fact, in our experience, control of supradiaphragmatic, intrapericardial IVC with subsequent thrombectomy without the need of sternotomy, as described by Ciancio et al. has been successfully done in a case of level III thrombus by this approach [15]. In one case of level III IVC thrombus with a long bland thrombus, we had a great deal of difficulty in getting control of infrarenal IVC and hence we had to make a double Makuuchi incision not only for tumor thrombectomy but also for IVC interruption.

The longer operative times [median: 290 (range: 180-970) minutes] and higher blood loss [median: 900 (range: 300-3800) mL] in our study are supported by the fact that there are seven cases of RCC with IVC thrombus and two cases of RCC with liver infiltration and blood loss in such cases with longer operative times is usually anticipated. 

## Conclusions

The MM incision provides excellent exposure to structures in the right upper abdomen for a wide variety of complex and advanced renal and adrenal pathologies mainly tumors. Also, urologists, cardiothoracic and liver surgeons can work through the same incision where renal/adrenal, major vascular and hepatobiliary/splenic pathologies are being dealt with simultaneously.
